# Does Bromelain-Cisplatin Combination Afford In-Vitro Synergistic Anticancer Effects on Human Prostatic Carcinoma Cell Line, PC3?

**DOI:** 10.31661/gmj.v9i0.1749

**Published:** 2020-08-29

**Authors:** Fatemeh Amini Chermahini, Elham Raeisi, Mathias Hossain Aazami, Abbas Mirzaei, Esfandiar Heidarian, Yves Lemoigne

**Affiliations:** ^1^Cellular and Molecular Research Center, Basic Health Sciences Institute, Shahrekord University of Medical Sciences, Shahrekord, Iran; ^2^Department of Medical Physics and Radiology, School of Allied Medical Sciences, Shahrekord University of Medical Sciences, Shahrekord, Iran; ^3^Clinical Biochemistry Research Center, Basic Health Sciences Institute, Shahrekord University of Medical Sciences, Shahrekord, Iran; ^4^Department of Cardiology and Cardiac Surgery, Kashani and Hajar University Hospitals, School of Medicine, Shahrekord University of Medical Sciences, Shahrekord, Iran; ^5^Department of Medical Physics, Institute for Medical Physics, Ambilly, France

**Keywords:** PC3 Cells, Bromelain, Cisplatin, Synergistic Effect, Clonogenic Cell Assay, p53 Gene

## Abstract

**Background:**

Bromelain enhances anticancer impacts to chemotherapeutic agents. The question as to whether bromelain does promote *in-vitro* cytotoxic and proapoptotic effects of cisplatin on human prostatic carcinoma PC3 cell line was investigated.

**Materials and Methods:**

PC3 (human prostatic carcinoma) cells were treated either single or in combination with bromelain and/or cisplatin. MTT, clonogenic assay, flow cytometry and real-time quantitative polymerase chain reaction were used to investigate cell viability, colony formation, proapoptotic potential and *p53* gene expression, respectively.

**Results:**

Cisplatin (IC_10_) combined with bromelain (IC_40_) significantly affected PC3 cell viability, inhibited colony formation, as well increased *p53* proapoptotic gene expression compared to cisplatin single treatment. Nevertheless, bromelain-cisplatin chemoherbal combination did not display any additive proapoptotic effect compared to single treatments.

**Conclusion:**

Bromelain-cisplatin chemoherbal combination demonstrated synergistic *in-vitro* anticancer effect on human prostatic carcinoma cell line, PC3, that drastically reduced required cisplatin dose.

## Introduction

Prostatic cancer is the second prevalent male malignancy. Sustained by prostatic anarchic epithelial cell proliferation, this type of cancer presents higher rates of cell proliferation with the advanced age, and portends a high propensity to metastasize [1]. Surgery, electrochemotherapy, radiotherapy, inhibiting hormone therapy, and chemotherapy comprise the mainstay of multi-disciplinary anticancer therapeutics [2,3]. Approaching a 5-year survival lower than 50%, the fate of this cancer is clouded by resistance to chemotherapy. Cisplatin, a platinum-based chemical drug (cis-diamminedichloroplatinum or CDDP) is widely used in various anticancer chemotherapy protocols, such as in the case of ovarien, cervical and prostatic cancer [4]. As a restrictive clinical issue, serious cisplatin side effects (nephrotoxicity, myelosuppression, ototoxicity, anaphylactic reactions, peripheral neuropathies, and hypomagnesemia) can jeopardize the therapeutic pursuit, inflicting substantial reduction in recommended therapeutic drug titrations [5]. In view of alleviating side effects, enhancing clinical tolerance, and boosting anticancer properties of chemotherapy, it was sought to take advantage from combination of natural herbal products to chemical drugs [6,7]. Previous studies reporting on chemoherbal anticancer combination indicated encouraging results. Extricated from pineapple (Ananas comosus), bromelain is a cysteine proteinase distinguished to display *in-vitro* anticancer properties such as inhibition effects on cell proliferation, as well apoptotic potential [8]. Bromelain as a mixture of proteases, has long been used in natural medicine across the globe. Anti-inflammatory, fibrinolitic, immunomodulatory and anticancer properties of bromelain were reported aside its classical beneficial effects on digestion, debriding burn scars and promoting action on wound healing process [9]. The bromelain anticancer properties were reported by a few clinical evidences; yet, mainly investigated by *in-vivo *animal models as well *in-vitro* studies in a wide array of animal or human cancer cell lines [10]. Different mechanisms to bromelain anticancer potency were advanced. Pertaining to observed feature as to inhibit cell proliferation, undermine invasiveness, and promote apoptosis to neoplastic cells, a more complex and intricated anticancer mechanism of action involving multiple pathways was assigned to bromelain, which signalizations remain the focus of ongoing researches cells [11]. This study was designed to investigate the *in-vitro* bromelain anticancer effects alone or in chemoherbal combination with cisplatin on human prostatic carcinoma cell line, PC3.

## Materials and Methods

###  Human Prostatic Carcinoma, PC3, Cell Culture

PC3 human prostatic carcinoma cell line was purchased from the Pasteur Institute (Tehran/ Iran). Cells were grown using Roswell Park memorial Institute (RPMI)-1640 (Life Technologies, California, USA) as the culture medium, supplemented with heat-inactivated 10% FBS, 1% antibiotics (100 U/ml of penicillin and streptomycin) (Life Technologies, California, USA) at 37°C, 98% humidity, under 5% CO_2_ partial pressure. Cells were trypsinized and passaged prior reaching cellular confluence.

###  Bromelain and Cisplatin Stock Solution Preparation

 Bromelain and cisplatin were sourced from Merck (Merck CO., Darmstadt, Germany). The stock solution to bromelain and cisplatin was prepared by dilution using sterile deionized water. Stock concentrations for bromelain and cisplatin were 30µM and 250µM, respectively. The stock solutions were kept at 4°C and away from lightening. Subsequent required concentrations were obtained by diluting stock solution with RPMI.

###  Single Treatment 

Each well of 96-well plates was filled with 5×10^3^ cells per 200µL from PC3 cell culture medium. The well plate was incubated overnight under standard protocol. Subsequently, cells were treated with different concentrations of bromelain (0 to 30 µM) or cisplatin (0 to 100µM) [12]. Cells were incubated at three incubation times: 24, 48, and 72 hours. Each single treatment was repeated three times in three independent experiments.

###  Bromelain-Cisplatin Chemoherbal Combinational Treatment

Wells filled and incubated with PC3 cell line were treated by four distinct combinational concentrations of bromelain and cisplatin as follow: I) bromelain IC_10_ plus cisplatin IC_40_, II) bromelain IC_20_ plus cisplatin IC_30_, III) bromelain IC_30_ plus cisplatin IC_20_, IV) bromelain IC_40_ plus cisplatin IC_10_. The treated cell were kept incubated for 48 hours. The chemoherbal combinational treatment was repeated three times in three independent experiments.

###  MTT Assay to Appraisal Cell Proliferation Inhibitory Effect

Assessment of PC3 cell proliferation inhibitory effect to single or combinational treatments relied on MTT assay [3-(4,5-dimethylthiazol-2-yr)-2,5-diphenyl tetrazolium bromide]. To the latter end, MTT solution (5mg/mL) was added to wells followed by a mandatory 4-hours incubation. Cells were dissolved in 200 µL of dimethyl sulfoxide (DMSO) (Merck) that was incubated for 20 minutes. Optical density of each well was measured using a microplate reader (Stat Fax-2100, Awarness Technology Inc, Florida, USA) on the light-range of 490-570 nm [13].

###  Clonogenic Cell Assay

PC3 cells were seeded in 6-well plates (100 PC3 cells per well) and incubated overnight. Cells were treated by single bromelain/cisplatin, or bromelain-cisplatin chemoherbal combination, and kept incubated for 48 hours. To form cell colonies, previously treated PC3 cells were incubated for 14 days (37°C, 98% humidity, under 5% CO_2_ partial pressure) and the culture medium (RPMI) was renewed every 48 hours. Visible colonies were fixed with 70% ethanol and stained with 0.5% crystal violet to measure plating efficiency (PE). Cell culture efficiency was measured by the formula [yield efficiency=(number of colonies formed / number of cultured cells) × 100]. The survival ratio for treatment groups was calculated using the formula [survival ratio=(efficiency of cultures with treatment / efficiency of cultures without treatment) × 100) [14].

###  Annexin V-PI Staining to PC3 Cell Apoptosis Assay

PC3 cells were seeded in 6-well plates (2×105^5^cells per well) and incubated for 24 hours. Reaching 75% cell confluence incubated PC3 cells were treated with bromelain, cisplatin, and bromelain-cisplatin chemoherbal combination for an incubated time of 48 hours. Dead or viable cells were trypsinaized, washed with phosphate-buffered saline (PBS), and stained with annexin (BD Bioscience California, USA) for 30 minutes at room temperature. Cell analysis was performed using a flowcytometer (CYFLOW space, Patrick, Germany) according to the manufacture′s protocol [13].

###  Real-Time Polymerase Chain Reaction (PCR)

PC3 were seeded at a density of 6×105^5^cells/well for 24 hours, and exposed to bromelain-IC40, cisplatin-IC10, and breomelain-IC40 plus cisplatin-IC10 combination. In accordance to manufacture′s instructions, PC3 cells were harvested and lyzed with RotiZOL. Determination of mRNA concertation to each single or combinational treatment was undertaken using Nanodrop spectrophotometer (Thermo, USA). Reverse transcriptase was used to synthetize cDNA from 1 µg of total mRNA pool which was carried out using Prime Script^TM^ reagent Kit (Takara Bio Inc., Japan) for cDNA synthesis [13]. Real-time quantitative polymerase chain reaction (RT-qPCR) expanded cDNA using SYBR® Green PCR Master Mix (Takara Bio Inc., Japan) in the presence of specific primers for *p53 *(forward: 5′-CCCATCCTCACCATCATCACAC-3′; reverse: 5′-GCACAAACACGCACCTCAAAG3′), and, the *glyceraldehyde -3 -phosphate dehydrogenase (GAPDH) *(forward: 5′ACACCCACTCCTCCACCCTTTG3′; reverse: 5′GTCCACCACCCTGTTGCTGTA-3′). The primers (Eurogentec Seraing, Belgium) were designed using Oligo 7.0 software (Molecular Biology Insights, Cascade, Co, USA) and confirmed by the blast (NCBI). Rotor-Gene 3000 (Corbett, Australia) was used to detect expression of *p53* gene for each of the concentrations. The temperature profile for the reaction was an initial denaturation stage of 95°C at 10 min, and then a three-step program was developed for 40 cycles including 95°C for 10 S, 62°C for 15 S, and 72°C for 20 S, respectively. *GAPDH*, a housekeeping gene, was amplified separately for normalizing the data.

###  Data Processing

Data were obtained from 3 independent repetitions to each single or combinational treatment, data analysis was undertaken using SPSS (version 20, Inc, Chicago, IL, USA) and GraphPad Prism 6 (GraphPad *Software* Inc., San. Diego, CA, USA). Data are expressed as means±standard error (SE). Cell viability percentages were plotted against different bromelain and cisplatin concentrations, and their respective IC_10_, IC_20_, IC_30_, IC_40_, IC_50 _were calculated by regression prohibit. Combination index values (CI) were estimated using CompuSyn software (Combo SynInc, City, State, USA). Synergistic, additive, and antagonist effects were respectively defined by CI<1, CI=1, and CI>1. Means comparison was preceded by Kruskal-Wallis test and Dunn’s. Statistical significance was defined as P<0.05.

## Results

###  Cell Proliferation Inhibitory Effect of Single Treatment

 PC3 cells were treated as for different incubation times of 24, 48, and 72 hours with bromelain or cisplatin as a single treatment. Concentration ranges to single treatment for bromelain and cisplatin were 0, 0.5, 1, 2, 3.5, 7, 14, and 28 µM and 0, 0.1, 1, 5, 10, 20, 40, 50, and 100 µM, respectively.

###  Bromelain Single Treatment

**Figure 1:**
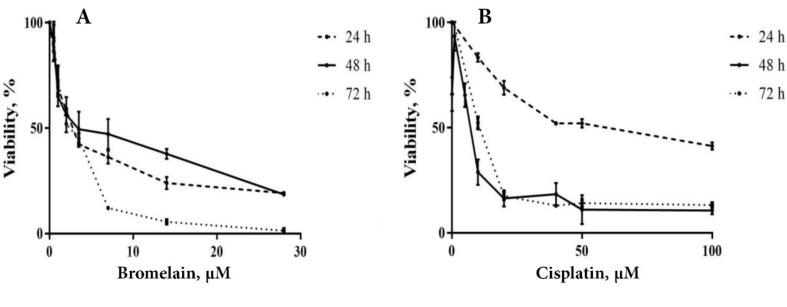
PC3 Cell viability after treatment with a various concentration of (A) bromelain and (B) cisplatin for 48 hours. Data are expressed as mean±SE from 3 independent repetitions.

At three different incubation times, bromelain displayed significant cell inhibitory effect on PC3 cell viability in a dose and time-dependent manner (P<0.05) (Figure-1). Bromelain 50% inhibitory concentration (IC50) was obtained for each of the three different incubation times of 24, 48, and 72 hours as 3.5 µM, 4.4 µM, and 0.8 µM respectively. *Cisplatin single treatment*: At three different incubation times, cisplatin displayed significant cell inhibitory effect on PC3 in a dose and time dependent manner (P<0.05) (Figure-1). Cisplatin 50% inhibitory concentration (IC50) was obtained for each of the three different incubation times of 24, 48, and 72 hours as 55.02 µM, 3.09 µM, and 10.5 µM respectively.

###  Cell Proliferation Inhibitory Effect of Chemoherbal Combinational Treatment

**Figure 2:**
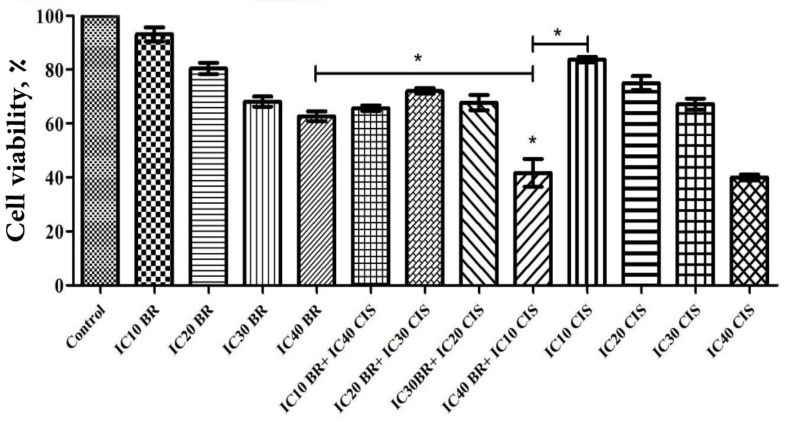
PC3 cell viability after treatment with IC_10_, IC_20_, IC_30_, IC_40_ of bromelain and cisplatin as a single or combinational treatment for 48 hours. Data are expressed as mean±SE from 3 independent repetitions.

Considering the obtained respective values of IC_50_ to each of the bromelain or cisplatin single treatments, the incubation time of 48 hours was chosen to conduct chemoherbal combinational treatment. The latter was undertaken at 4 different combinational concentrations as to obtain an additive IC score of 50 (Table-1). The combination IV displayed significant inhibitory effect in comparison to the control and single treatment either with bromelain or cisplatin (Figure-2).

###  Effect of Chemoherbal Combination Treatment on PC3 Colony Formation

**Figure 3:**
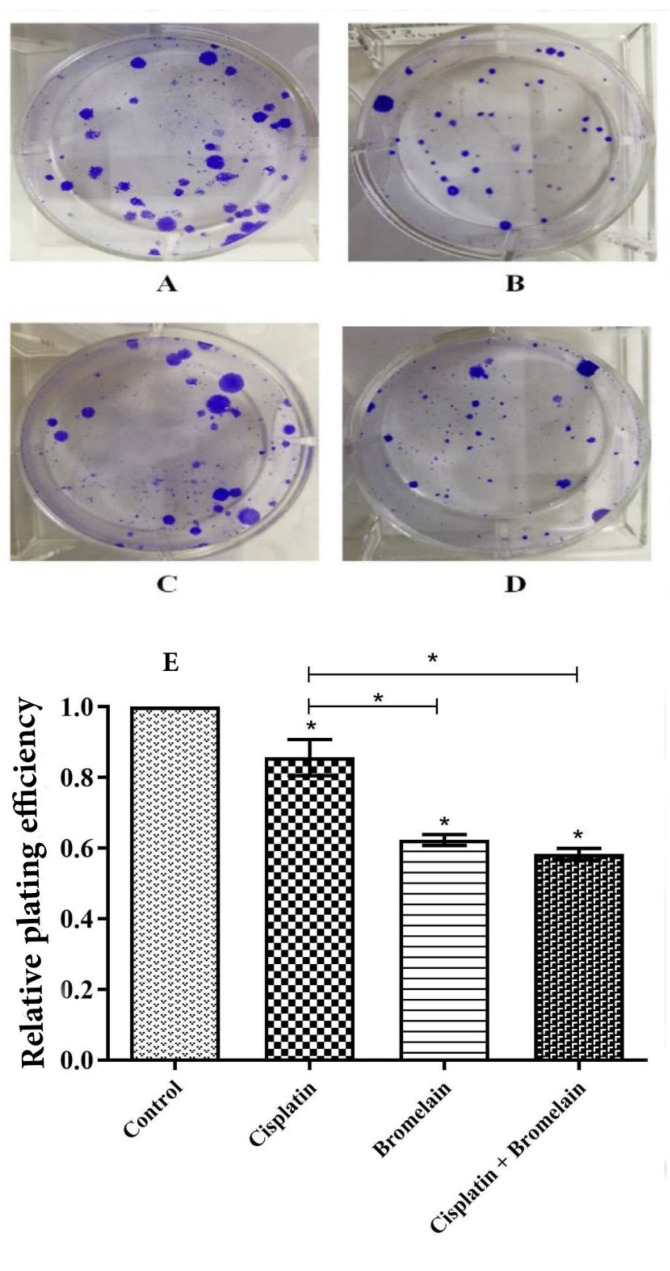
Colony formation (A) untreated, (B) treated with 2.5 µM bromelain, (C) treated with 1.5 µM cisplatin, (D) treated with 2.5 µM bromelain and 1.5 µM cisplatin and (E) relative plating efficiency. Data expression: mean±SE.

According to the results of combinational treatment on cell proliferation inhibitory, the combination IV was applied to clonogenic assay. The single treatments with bromelain IC_40 _and cisplatin IC_10_ were simultaneously investigated (Figure-3). The combination IV was significantly effective on clonogenic inhibition when compared to untreated control PC3 cells (P<0.05) and single cisplatin treatment (P<0.05). There was no additive effect of combination IV compared to single bromelain treatment.

###  Effect of Combinational Chemoherbal Treatment on PC3 Apoptosis Induction

**Figure 4:**
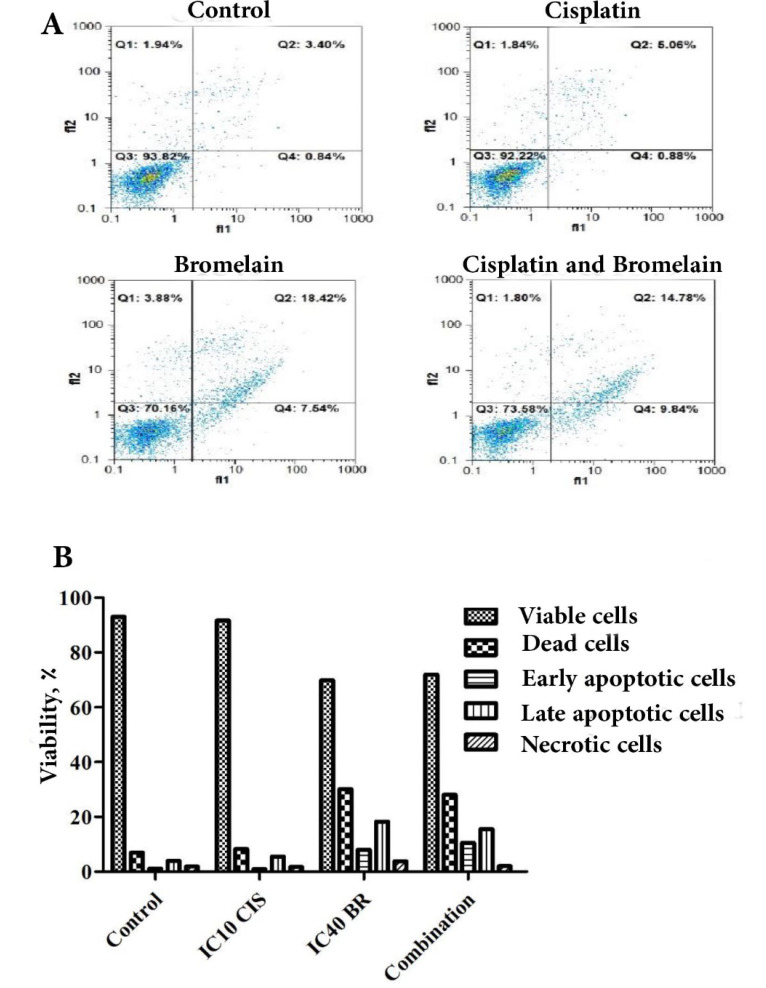
(A) Analyzing annexin V-FITC/PI by Flow Cytometry, (B) total apoptosis cells treated with bromelain (2.5 μM), cisplatin (1.5 μM) or combination of bromelain (2.5 μM) plus cisplatin (1.5 μM) for 48 hours. Data expression: mean±SE.

 Chemoherbal combination IV was applied to assess the chemoherbal effect on PC3 apoptosis induction compared to the control and single treatment with cisplatin or bromelain. Chemoherbal combination resulted in significant apoptosis enhancement compared to the control PC3 (P<0.05). Chemoherbal combination effect on PC3 apoptosis induction did not reach significant difference compared to each of the single treatments (Figure-4).

###  Effects Combinational Chemoherbal Treatment on PC3 Apoptosis-Related Gene Expression 

**Figure 5:**
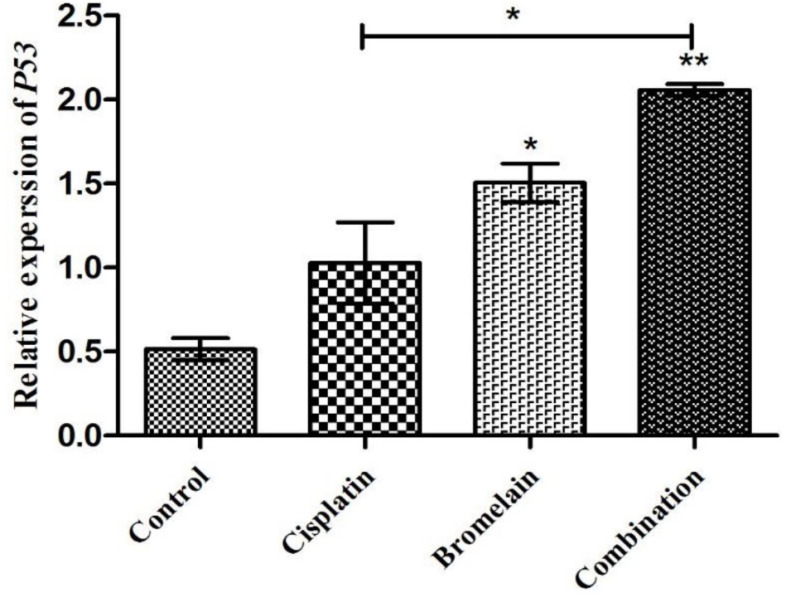
Changes in *p53* gene expression treated with bromelain (2.5 μM), cisplatin (1.5 μM) or combination of bromelain (2.5 μM) plus cisplatin (1.5 μM) for 48 hours. Data expression: mean±SE (* P<0.05, ** P<0.01).

Chemoherbal combination IV was investigated in parallel with control PC3 and single treatment with bromelain or cisplatin (Figure-5). Chemoherbal combination resulted in significant higher expression of proapoptotic *p53* gene compared to the control (P<0.01), as well with cisplatin single treatment (P<0.05).

## Discussion

Behind the lung cancer, the prostatic neoplasm represents the second prevalent human male malignancy. The therapeutic armantarium against human prostatic cancer encompasses chemotheraphy, radiotherapy, brachytherapy, electro-chemotherapy, targeted therapy and surgery [3,15]. Despite continuous effort and achievements witnesed in the last 4 decades, the prostatic anticancer therapy is fraught with a failure rate reaching 30 to 50%. As a pillar to prostatic anticancer therapy, chemotherapy aims to inhibit cancer cell proliferation, promoting apoptosis induction and increase radiosensitivity of the neoplastic cells. Chemotherapeutic agents effect through various mechanisms of action as to reduce the tumor size and increase tumor vulnerability [16]. In practice, the effective conduct of chemotherapy can nevertheless be bared by developing resistance to the chemotherapeutic agents, as well their attendant side-effects jeopardizing their clinical tolerance [17]. Occurence chemotherapy related side-effects accounts for sustanial morbidity and mortality [18]. Seeking to overcome chemotherapy limitations, chemoherbal combinational treatment (CHCT) emerged as a potential solution by taking in advantage the inate anticancer properties of natural herbal medicine to chemotherapeutic agents. Rutin, curcumin, thymoquinone, ZnO nanoparticles and bromelain recently proved their potency to enhance cytotoxicity of chemotherapeutic agents on various human cell lines [7,10,13,19-21]. Of recent, the synergistic or permissive anticancer potency of natural herbal products, reported by *in-vitro* investigations or animal models on human neoplastic cell lines, do provide new insights as to consider means and ways to modulate chemotherapy condut. The latter aimed to enhance clinical tolerance by lessening the required therapeutic dose of chemotherapeutic agents (chemoprotective effect), while boosting their anticancer potency by activating new complementary mechanisms or potentialising chemotherapeutic impact in a multi-modal course of actions [8-11]. The bromelain anticancer action through its anti-inflammatory property was suggested first by preventing tumorogenesis sustained by the process of chronic inflammation, hence considered being of chemopreventive potential [22]. Bromelain chemopreventive effects on human skin and colon tumorogenesis was reported [23]. Downregulation of NF-κB, Cox-2, and PGE2 in parallel with upregulaion of INF ᵞ, THF-α, IL-1ß and IL-6 were demonstrated as main bromelain anti-inflammatory modulatory pathways. Bromelain attributed anti-inflammatory modulation (NF-κB, Cox-2, and PGE2 downregulation) is considered to hindern neo-anginogenesis required to tumor progression. Further, the proteolytic properties of bromelain was advocated as to be the leading mechanism of its anticancer action [8]. Accordingly, bromelain displayed blocking cell adhesion, inhibiting cell migration and invasiveness on glioblastoma [8,11,24-27]. The bromelain inhibitory effects on neoplastic cell proliferation (cytostatic or cytotoxic) were widely documented on various animal or human neoplasms such as leukemia, lymphoma, sarcoma, melanoma, lung carcinoma, gastric-intestinal carcinoma, gliolastoma, breast cancer, epidermoid carcinoma, melanoma and malignant mesothelioma [16,28,29]. Chang *et al*. demonstrated that bromelain increased oxidative stress and superoxide production by 6-fold in bromelain treated human colorectal cancer cells as part of its anti-proliferative mechanisms [27]. The latter study also reported on *in-vitro* bromelain effect on activation of macrophagy pathway and lysosome formation through increasing levels of autophagy-related proteins (ATG5/12, beclin, p62, and LC3I/II) leading to programming cell death. The microenviromental anti-inflammatory properties of bromelain by uncoating cancer cells (depolymerizing MUC-1,fibrin and albumin) is thought to enhance tumor exposure to the host defense by increasing lymphocyte-to-tumor adhesion [30]. Bromelain showed be capable of counteracting MUC-1 onco-glycoprotein overexpression that should impart enhanced proliferation and antiapoptotic feature to cancer cells along with conferring invasiveness and chemoresistance to various human tumors. The aptitude of bromelain to engage *in-vitro* human leukemia cell differentiation was already mechanisms reported. Additionnaly, bromelain proved to be of radiosensitizing feature in 4T1 mouse breast cancer cell line [14]. In the current study, single-agent bromelain or cisplatin treatment displayed a significant dose and time-dependent effect on inhibition of PC3 cell proliferation at three different incubation times of 24, 48 and 72 hours (Figure-1). Unaminously, the previous reports pointed out the dose and time–dependant feature of bromelain mode of action on various cancer cell lines. In our study, we showed bromelain induced the cell proliferation reduction, which is in agreement with those previous studies [29,31]. Bromelain-cisplatin CHCT effects on PC3 cell proliferation was studied at four different combinations with an additive IC score of 50% at 48 hours incubation time (Table-1). The CHCT combination-IV (bromelain IC_40 _plus cisplatin IC_10_) was the only combinational treatment displaying a significant additive effect on inhibition of PC3 cell proliferation in comparison to the single-agent treatment with bromelain or cisplatin (Figure-2). The latter resulted in a 38 time lesser cisplatin dose compared to cisplatin IC_50 _alone as to inhibit PC3 cell proliferation. Pillai *et al*. showed that bromelain-cisplatin CHTC increased a significant cytotoxic effect on malignant peritoneal mesothelioma (PET and YOU cells) compared to cisplatin single-agent [32]. Similary, Pauzi *et al*. demonstered *in-vitro* synergistic effect of bromelain-cisplatin CHCT on human breast MDA-MB-231 cancer cells. Using a combinational concentrain of bromelain 2 μM plus cisplatin 1.5 μM, the authors concluded a 16 time reduction in cisplatin dose compared to its IC_50_ dose [12]. These results are in line with the findings of present study, however, indicating the PC3 cell proliferation may be more sensitive to bromelain-cisplatin CHTC compare to MDA-MB-231 cancer cells. When compared to the control and cisplatin single-agent treatment, the additive effect of combination-IV (bromelain IC_40_ plus cisplatin IC_10_) on PC3 cell inhibition to colony formation was noticed (Figure-3). Colony formation of PC3 cells was inhibited significantly with bromelain-cisplatin combination compared to the cisplatin single-agent treatment; nevertheless, without significant difference when compared to the bromelain single-agent treatment. Nasiri *et al*. demonstrated the clonogenic inhibitory effect of bromelain as a single-agent or in combination with superparamagnetic iron oxide nanoparticles (SPIONs) on human cervical cancer cell (Hela), human breast cancer (MDA-MB-231), and murine breast cancer (4T1). Our finding is in agreement with this previous study [33]. In the current study, bromelain as a single-agent or combination with cisplatin decreased the percentage of plating efficiency of PC3 cells (Figure-3). We found the similar results as the previously reported studies showing reduction in plating efficiency of treated human cancer cells with cisplatin as a single agent [34] or bromelain as a single-agent or in combination with SPIONs [33]. In the current *in-vitro* study, although CHCT resulted in higher expression of proapoptotic *p53* gene (compared to control PC3 cells, bromelain or cisplatin single-agent treatments), the latter did not result in any additive synergistic effect on promoting cell apoptotic induction using flow cytometry (Figure-4). The latter converged to the findings reported by Pauzi *et al*, investigating bromelain-cisplatin CHCT on human breast MDA-231MB cancer cells, whereby the authors discribed synergistic effect on cancer cell apoptosis compared to single-agent treatment [12]. Nevertheless, bromelain-cisplatin CHCT showed activating multiple apoptotic pathways (signalization cascade) compared to single-agent treatment. Reducing expression of antiapoptotic regulators such as cIAP 1, Bcl-2, catalase,clusterin, HO-1, livin, XIAP, HSP27 and claspin was also demonstrated in human breast cancer line [12]; the latter in support of mithochondrial apoptotic pathway using bromelain-based CHCT. In the current study, expression of *p53* proapoptotic protein based on the results of RT-PCR was significantly increased using CHCT (combination-IV; bromelain IC_40_ plus cisplatin IC_10_) compared to each of the single-agent treatments (Figure-5). Upregulation of *p53* was observed with the chemoherbal combination-IV (bromelain IC_40_ plus cisplatin IC_10_). *P53* upregulation lead to higher PC3 cell death using the combination-IV. We found similar findings to those previously reported studies, showing upregulation in *p53* of treated human cancer cells with bromelain as a single-agent treatment [29,35] or in combination with cisplatin [12], n-acetylcysteine [36] or SPIONs [33]. Other studies demonstrated increased expression of proapoptotic proteins such as HSP60 with bromelain-based CHCT. Overexpression of HSP70 known as a cytoprotective protein was also demonstrated as part of bromelain-based CHCT ability to induce autophagy [37]. In addition bromelain-based CHCT demonstrated increasing intracellular concentration of chemotherapeutic agents such as 5-FU as part of its presumed permissive effects on chemotherapy [32].

## Conclusion

Anticancer chemoherbal combinational treatment drives promising insights based on *in-vitro* and *in-vivo* reported investigations. Anticancer action of bromelain involves modulating different pathways that does depend on the cancer cell type and the experimental protocol. In the current study, bromelain as single-agent or in chemoherbal combinational treatment with cisplatin displayed anticancer properties on human prostatic carcinoma PC3 cell line, permetting to drastically reduce the *in-vitro* cisplatin dose. Taking together the available data to the anticancer features of bromelain and advocated multiple modulatory pathways, it is incentive to pursuit the research as to fully elucide the reproducibility of the reported results *in-vitro*, as well extending *in-vivo* studies on each type of human cancer cell lines.

## Acknowledgement

 The authors would express their grateful toward the staff of clinical biochemistry research center for their excellent dedication for conducting technical steps required to the current study as well the deputyship of Research and Innovation of Shahrekord University of Medical Sciences (Grant No. 2695) for their logistic support. This article is part of MSc thesis of Ms Fatemeh Amini-Chermahini.

## Conflict of Interest

 None.

**Table 1:** The Value of CI (Combination Index) of PC3 Cell Line Treated with Combination of Bromelain and Cisplatin after 48 h Incubation. CI<1: Synergistic Effect, CI=1: Additive Effects and CI>1: Antagonistic Effect.

**Combination Number**	**Dose Combination (µM)**		**CI **
	**Bromelain (IC), µM**	**Cisplatin (IC), µM**	
**I**	0.26 (IC_10_)	1.5 (IC_40_)	-
**II**	0.69 (IC_20_)	0.71 (IC_30_)	-
**III**	1.3 (IC_30_)	0.28 (IC_20_)	-
**IV**	2.5 (IC_40_)	0.08 (IC_10_)	0.59
